# Supplementary figures and images for: Subversion of phosphorylated SR proteins by enterovirus A71 in IRES-dependent translation revealed by RNA-interactome analysis
Source: PLoS Pathog. 2025 Jun 16;21(6):e1013242. doi: 10.1371/journal.ppat.1013242 (PMC12193706; doi:10.1371/journal.ppat.1013242)

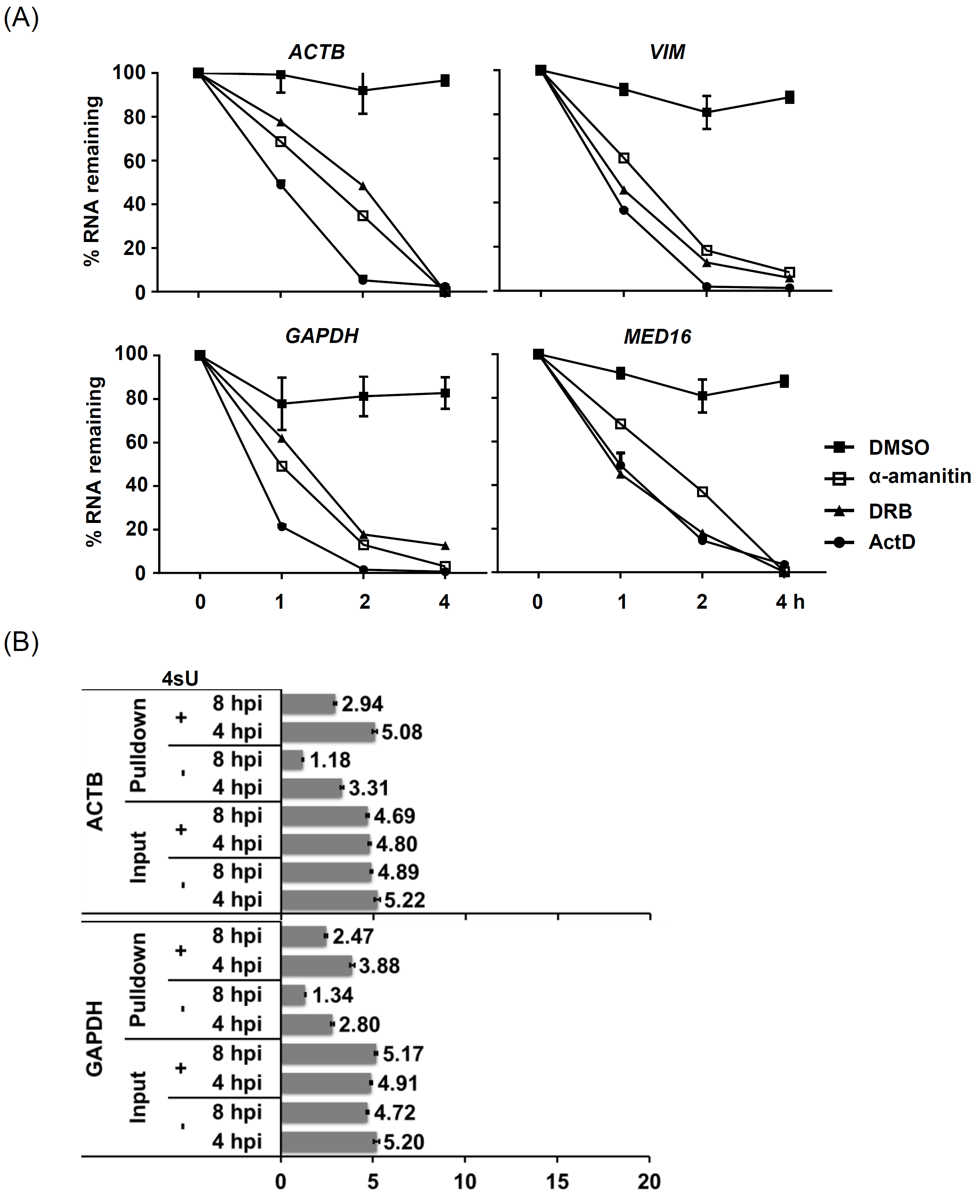

Supplement: S1 Fig — (A) Rhabdomyosarcoma (RD) cells were treated with 4 μM actinomycin D (ActD), 100 μM 5,6-dichlorobenzimidazole 1-β-D-ribofuranoside (DRB), or 2 μM α-amanitin for 4 hours. Reverse transcription-quantitative polymerase chain reaction (RT-qPCR) was performed using total RNA collected at the indicated time points. mRNA levels are expressed relative to the 0-hour time point (defined as 100%) and presented as the mean ± standard deviation (SD). Data shown are representative of three biologically independent experiments. (B) Total RNA isolated from RD cells treated with 2 μM α-amanitin, in the presence or absence of 4sU, was subjected to oligo(dT) pulldown at 4 or 8 hours post infection (hpi). RT-qPCR analysis of pulldown samples was normalized to 5% of the corresponding input RNA. Data represent the mean ± SD from three technical replicates of a single independent experiment. (TIF) [file ppat.1013242.s001.tif]

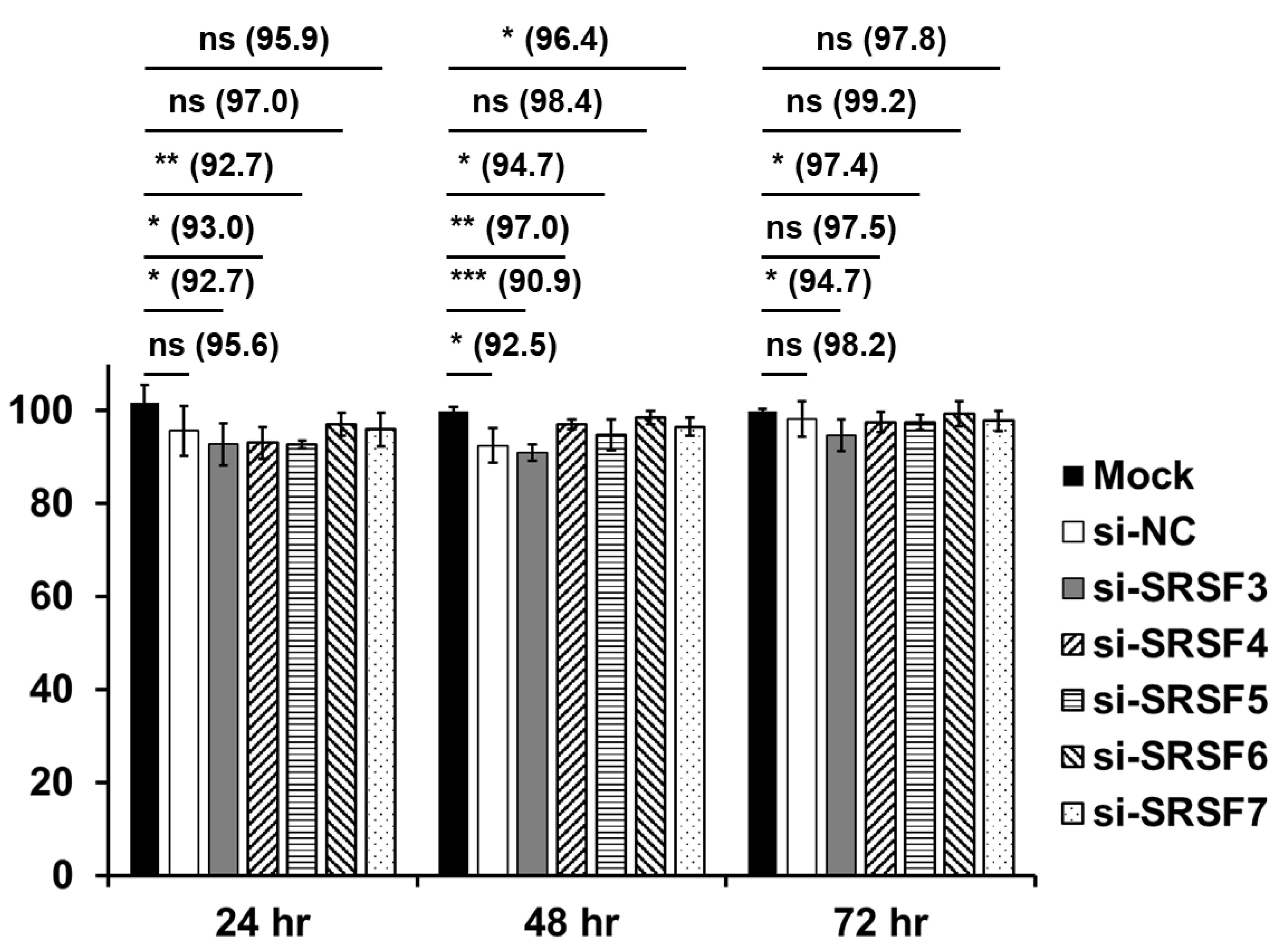

Supplement: S2 Fig — (A) MTS assay evaluating the cytotoxicity of knocking down SR proteins as indicated by the corresponding siRNA of each SR protein. RD cells were transfected with 100 nM siRNA for 24, 48, and 72 hours. Data are presented as the mean ± SD from three technical replicates of an independent experiment. Statistical analysis was performed using the Student’s t-test. ***: p < 0.001; **: p < 0.01; *: p < 0.05; ns: not significant. (TIF) [file ppat.1013242.s002.tif]

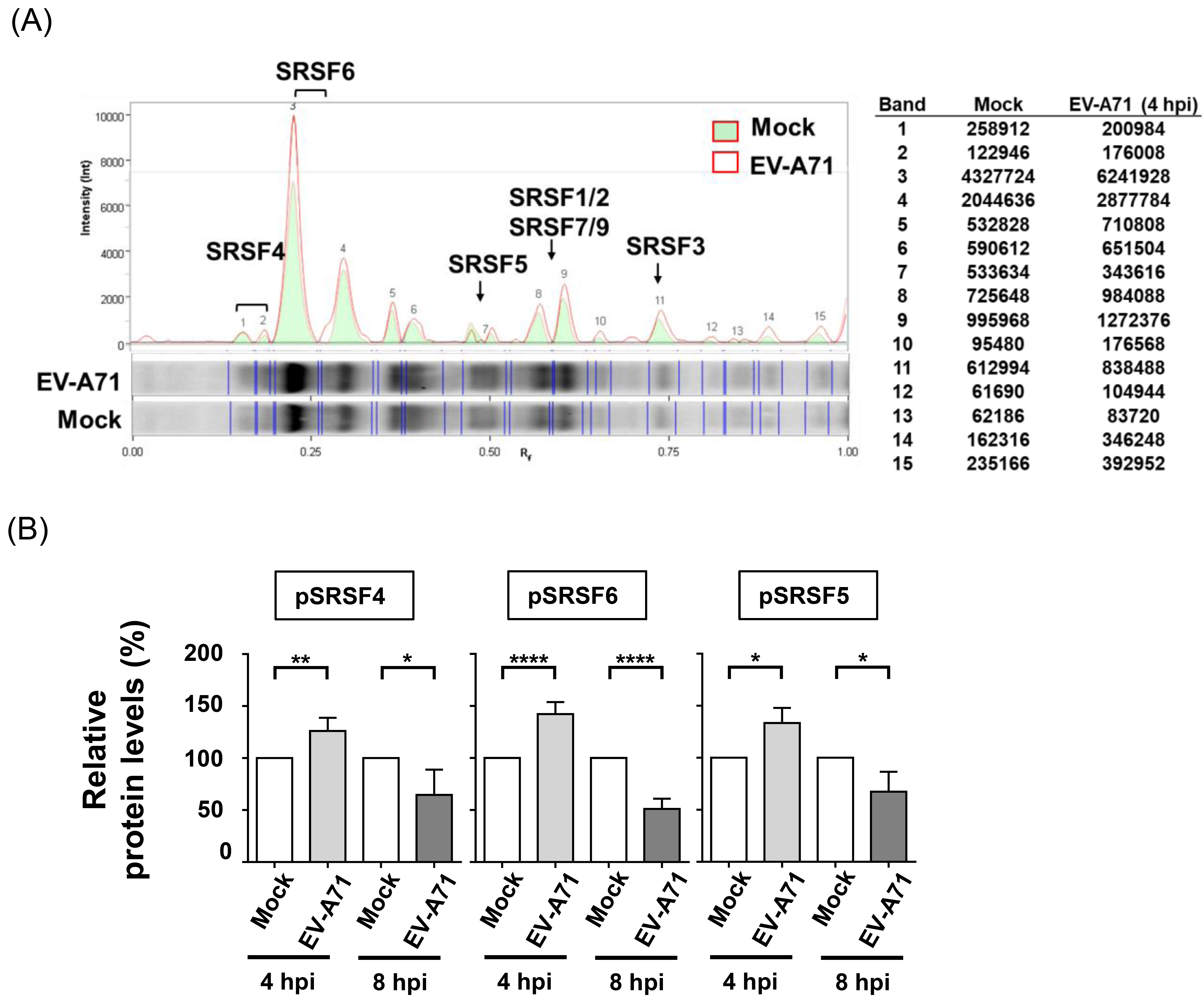

Supplement: S3 Fig — (A) The intensity plot (top panel) shows the migration profiles from mock-infected (red line) and EV-A71-infected (green shaded) RD cell lysates separated by Phos-tag SDS-PAGE. Each peak corresponds to distinct SR protein isoforms, with major proteins (e.g., SRSF1/2, SRSF3, SRSF4, SRSF5, and SRSF7/9) identified based on known migration patterns. Cropped gel images (bottom panels) are aligned with the intensity plot, and blue vertical lines mark the quantified gel regions. Relative mobility (Rf values) is indicated on the x-axis. (B) Quantitative analysis of signals corresponding to phosphorylated SRSF4, SRSF5, and SRSF6 (pSRSF4, pSRSF5, and pSRSF6, respectively). Signal intensities were quantified from digital images acquired with the ChemiDoc Imaging System (Bio-Rad, Hercules, CA, USA). Only non-saturated images were used to ensure accurate quantification. Band intensities were normalized to GAPDH, and protein levels in the mock group were set to 100%. Data are presented as mean ± SD from three independent experiments. Statistical analysis was performed using Student’s t-test. ****p < 0.0001; **p < 0.01; *p < 0.05. (TIF) [file ppat.1013242.s003.tif]

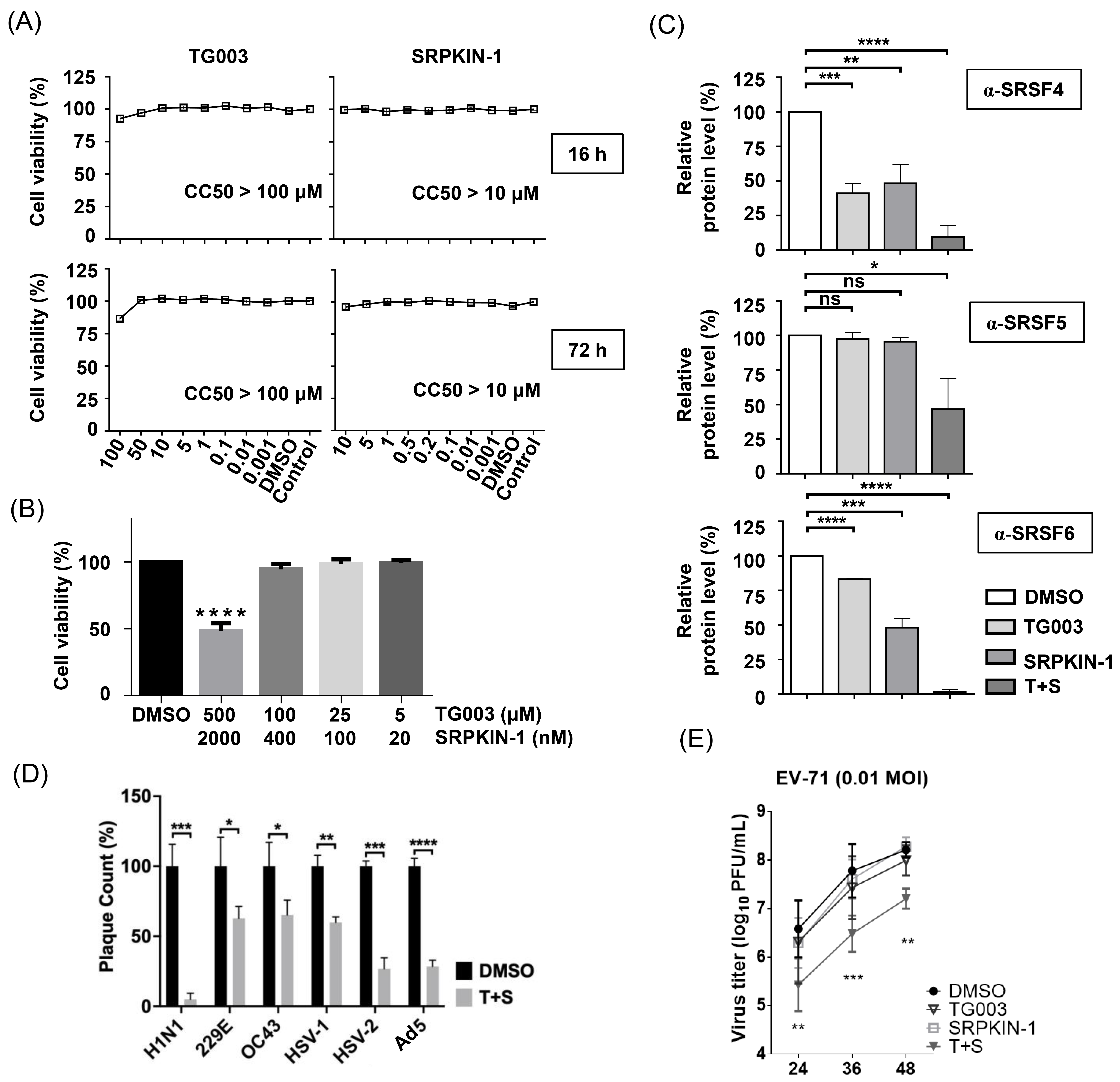

Supplement: S4 Fig — (A) MTS assay evaluating the cytotoxicity of TG003 and SRPKIN-1 in RD cells at various concentrations. The 50% cytotoxic concentration (CC50) of TG003 and SRPKIN-1 was determined to be ≥ 100 μM and ≥10 μM, respectively. (B) Combinations of TG003 and SRPKIN-1 at different concentrations, based on the CC₅₀ values in (A), were tested for their combined effects on RD cell viability. (C) Effects of various inhibitors on the phosphorylation levels of SRSF4, SRSF5, and SRSF6. Protein levels were quantified by normalizing to GAPDH, with the DMSO group set as 100%. Data represent the mean ± SD from three independent experiments. (D) Plaque reduction assays evaluating the antiviral effects of TG003 and SRPKIN-1 against influenza virus H1N1, coronaviruses 229E and OC43, and DNA viruses HSV-1, HSV-2, and adenovirus serotype 5 (Ad5). Data represent the mean ± SD from three independent experiments. (E) Growth curve analysis of EV-A71 replication at low MOI under various treatment conditions, as indicated by the respective symbols. A significant reduction in EV-A71 replication was observed with combined TG003 and SRPKIN-1 treatment (T + S, represented by inverted triangles). Statistical analysis for (B), (C), and (D) was performed using Student’s t-test; and for (E) using two-way ANOVA. ****: p < 0.0001; ***: p < 0.001; **: p < 0.01; *: p < 0.05; ns: not significant. (TIF) [file ppat.1013242.s004.tif]
