# Supplementary material for: Subversion of phosphorylated SR proteins by enterovirus A71 in IRES-dependent translation revealed by RNA-interactome analysis
Source: PLoS Pathog. 2025 Jun 16;21(6):e1013242. doi: 10.1371/journal.ppat.1013242 (PMC12193706; doi:10.1371/journal.ppat.1013242)
Supplement: Attachment — Submitted filename: 20241223 Rebuttal letter.docx [file ppat.1013242.s009.docx]

December 22th, 2024

Peter Sarnow

Academic Editor

*PLOS Pathogens*

Dear Dr. Sarnow and the Editorial Team,

Thank you for the opportunity to submit our manuscript, "Subversion of Phosphorylated SR Proteins by Enterovirus A71 in IRES-Dependent Translation Revealed by RNA-Interactome Analysis," for consideration at *PLOS Pathogens* **(PPATHOGENS-D-24-00744)**. We greatly appreciate the thoughtful feedback from reviewers #2 and #3, which has been invaluable in refining our work.

We acknowledge the concerns raised regarding the lack of controls and alternate interpretations. In response, we have made substantial revisions to address these issues, including the addition of key controls, knockdown experiments for SR proteins, and clearer experimental details. We have also expanded the manuscript to include more comprehensive data and discussions on the contributions of host and viral RNA, inhibitor efficacy, and the effects of SR proteins in EV-A71 infection.

We believe these revisions significantly strengthen our conclusions, and we hope the manuscript will now meet the high standards of *PLOS Pathogens*. We look forward to your feedback and are happy to submit this revised version for further consideration, referring to the original submission **(PPATHOGENS-D-24-00744)**.

Thank you for your time and consideration.

Sincerely,

Kuo-Ming Lee

International Master Degree Program for Molecular Medicine in Emerging Viral Infections, College of Medicine, Chang Gung University, Taoyuan, Taiwan

Emerging Viral Infections, College of Medicine, Chang Gung University, Taoyuan, Taiwan

Tel: +886-3-2118800 ext. 3727

Fax: +886-3-2118174

Email: [g39002007@mail.cgu.edu.tw](mailto:g39002007@mail.cgu.edu.tw)

We thank the reviewers and editors for their thorough evaluation of our manuscript, "Subversion of Phosphorylated SR Proteins by Enterovirus A71 in IRES-Dependent Translation Revealed by RNA-Interactome Analysis" **(PPATHOGENS-D-24-00744)**. We sincerely thank the reviewers and editors for their thoughtful and constructive comments on our manuscript. We have carefully addressed all the points raised, revising the manuscript to incorporate additional data, improve clarity, and address concerns about experimental design and interpretation. Below, we provide a detailed, point-by-point response to each comment, highlighting how we have revised the manuscript to address the reviewers' feedback. We hope these revisions strengthen the manuscript and align with the reviewers' and editors' expectations.

**Reviewer #1:**

The manuscript by Lee et al., reports a detailed study of the temporal RNA-protein interactome of Enterovirus-A71. The authors found significant changes in early and late phase of the viral RNPs composition using 4-thiouridine tagging of newly synthesized RNA followed by mass spectrometry analysis. Inhibition of cellular transcription was done using DRB, actinomycin-D and alpha-amanitin. Viral RNA synthesis and also virus titer was resistant to alpha-amanitin. In summary, the manuscript shows a temporal remodeling of the RNP interactome. They found a redistribution of phosphorylated SR proteins during the early phase of infection associated to the viral translation machinery. In contrast, the RNPs identified at the late phase of infection include cytoplasmic membrane-bound proteins. Polysome profiles indicated that phospho-SR proteins co-sediment with 80S monosomes at 4 h post infection. Treatment with inhibitors of SR protein phosphorylation partially inhibit EV-A71 IRES-dependent translation, consequently decreasing viral replication. The authors appropriately discussed the results, referring to as the lack of knowledge of the impact of individual SR proteins for viral RNA translation, and the need of future works to understand the role of SR proteins phosphorylation during EV infection. The conclusions are novel and supported by solid results, despite future work is needed to understand the mechanism of action of all these proteins. The manuscript is well written and of broad interest.

We thank Reviewer #1 for the thorough evaluation and positive feedback on our manuscript. We are delighted that the reviewer found our study novel, well-written, and of broad interest. We appreciate the acknowledgment of our findings, particularly the temporal remodeling of the RNP interactome and the role of phosphorylated SR proteins in EV-A71 IRES-dependent translation.

In response to the constructive feedback from all reviewers, we have made several revisions to improve the clarity and robustness of the manuscript. Specifically, we have incorporated additional quantitative analyses, clarified the description of experimental methods and results (e.g., revised Figures 3F and 7A-C), and provided an expanded discussion on the role of individual SR proteins and their phosphorylation states in viral RNA translation. These updates aim to address the limitations highlighted and ensure that the manuscript presents our findings as comprehensively as possible.

**Reviewer #2:**

Strengths- the work is ambitious in trying to define the RBPome of infection at different stages in the early phase of infection.

Weaknesses- the writing and presentation of some data needs improving. In the discussion the authors state that much of the data needs further investigation to determine its significance and that the interactomes identified may be contaminated by non viral interactors. The use of broad antibodies to the SR proteins makes it difficult to determine any specificity in function, the use of broad inhibitors raises similar problems. Clarity of data needs improving eg Figure 6A cannot be interpreted as presented.

The work is potentially novel and significant but without the follow up the authors have described it is unclear which of the list of interactors may have any relevance for replication. The authors don't discuss previous work on SR proteins and enterovirus replication, which is a big surprise.

We appreciate the acknowledgment of the ambition and novelty of our study in defining the RBPome at different stages of infection. We sincerely thank the reviewer for their constructive feedback, which has greatly helped us improve the quality and clarity of our manuscript. Below, we provide a summary response addressing the reviewer’s comments, with detailed explanations included in the accompanying point-by-point responses.

**Clarity of Data and Writing:** We have made significant efforts to improve the writing and presentation of the data, including addressing concerns about clarity and interpretability (e.g., revised Figure 7A from the original Figure 6A). The descriptions have been updated throughout the manuscript to enhance understanding.

**Broad Antibodies and Inhibitors:** We acknowledge the limitations of using broad-acting antibodies and inhibitors. To address these concerns, we performed additional targeted siRNA knockdown experiments and quantitative analyses to identify the specific roles of individual SR proteins and their phosphorylation status in EV-A71 replication (revised Figures 3F, 7B, and 7F).

**Relevance of Interactomes and SR Protein Function:** We have discussed the possibility of contamination in the RBPome interactome and have now carefully clarified the potential significance of our findings in the context of viral replication. We also expanded our discussion of previous work on SR proteins and enterovirus replication (e.g., SRSF3, SRSF4, SRSF5, and others) and highlighted their roles in IRES-dependent translation, as suggested by the reviewer.

While we acknowledge that some aspects of our findings require further investigation, our current results provide a solid foundation for understanding the regulatory roles of phospho-SR proteins in viral replication. We have explicitly stated the novel aspects of our work, including the role of phosphorylated SR proteins in IRES-dependent translation, and clarified how our study adds to the existing literature.

We believe the revisions address the reviewer’s concerns comprehensively. We appreciate the reviewer’s thoughtful comments and have provided detailed explanations and data to support our claims in the point-by-point responses.

**Major Issues: Key Experiments Required for Acceptance**

**Reviewer #2:**

1) The authors report SR family proteins in the EV71 RNA interactome. The authors use a broad acting SR protein antibody and broad acting phosphoSR protien antibody throughout this study and state that because they used this antibody they can't be sure which SR protein, if any may have a specific effect, not alone if any of those they identified have any role in EV71 replication. The use of broad acting inhibitors shows incomplete inhibition of SR protein phosphorylation, again this is read out with a broad SR antibody, no specific proteins are identified. The authors don't acknowledge that SR proteins not identified in their interactome may instead be important for replication. A targeted siRNA screen depleting specific SR proteins should be performed to identify which, if any, SR proteins and viral replication.

We appreciate the reviewer’s insightful comments, which help emphasize important aspects of our manuscript. In response, we have conducted additional experiments to better address the concerns raised. While our initial use of pan-SR and phospho-SR antibodies allowed us to detect multiple SR family proteins, we acknowledge that these broad antibodies do not allow us to pinpoint specific SR proteins that may be involved in EV-A71 replication. To address this, we performed a targeted siRNA knockdown screen, depleting SRSF3, SRSF4, SRSF5, SRSF6, and SRSF7. The results revealed that SRSF4, SRSF5, SRSF6, and SRSF7 are critical for EV-A71 replication, while only a minor, non-significant reduction in replication was observed upon SRSF3 knockdown **(see revised Figure 3F, lines 246-254)**.

Additionally, to provide more specific insights into the impact of kinase inhibitors, we performed quantitative western blot analysis, as suggested by Reviewer #3 **(revised Figures 4A-C and 7A-B)**. These experiments were repeated to ensure reproducibility, and digital images were acquired using a CCD camera for quantitative analysis, as detailed in the **Materials and Methods section (lines 650-655)**. The analysis revealed that phosphorylation levels were reduced to approximately 40% of control levels upon treatment with TG003 and SRPKIN-1 **(revised Figure 7B)**. Consistent with the knockdown results, phosphorylation levels of SRSF4, SRSF5, and SRSF6 were significantly reduced under these conditions **(Supplemental Figure 7C, lines 353-361)**.

We apologize for not initially considering the possibility that SR proteins not identified in our interactome could play a role in replication. Although we did not observe co-fractionation of SR proteins with replication organelles using discontinuous fractionation (updated Figure 5), we recognize that the conclusions drawn in the original manuscript were limited to the SR proteins detected by broad antibodies. We have now included a discussion on the potential roles of other SR proteins and the impact of phosphorylation/dephosphorylation in regulating replication **(lines 310-313 and 410-414)**.

2) The authors perform experiments to try and tie SR protein responsiveness to translation as the SR proteins were enriched at the 4 hr but not 8 hr timepoint. There is a considerable discrepancy in these data that the authors do not acknowledge. (A) the authors say that inhibition of phosphorylation is incomplete- from the gel shown and as annotated, I cannot interpret the degree of inhibition in any condition. (B) Inhibition of IRES activity in the T+S treatment column is the same as for the TG003 alone treatment but in (C) only the T+S treatment significantly lowers replication. This discrepancy is not acknowledged. Moreover, the authors use a plasmid to express their reporter before infection with EV71. Infection may impact transcription and nuclear pore complex function. Therefore, these experiments should be repeated with RNA transfection instead of plasmid DNA transfection.

We apologize for presenting inadequate data in the original manuscript. As addressed in point 1), we have repeated the experiments and presented the results in a quantitative manner **(revised Figures 7A-B)**. Additionally, we validated the reduction of viral proteins VP0 and 3CD, as well as the viral titer, under high multiplicity of infection (MOI) conditions **(revised Figure 7C)**. Further, we confirmed the impact of kinase inhibitors on IRES-dependent translation using a mono-luciferase assay, in which firefly luciferase reporter RNAs harboring the EV-A71 5’ IRES were transfected **(revised Figure 7F)**. Consistently, in high MOI conditions, we observed a pronounced effect when SRPKIN-1 and TG003 were combined across all experiments **(lines 353-361 and 377-381)**.

Although a significant reduction in viral titer was observed under high MOI conditions, the effect was less pronounced compared to the approximately one-log reduction observed at low MOI **(original Figure 7C, now moved to Supplemental Figure 3E)**. Under low MOI conditions, moderate reductions were also observed with individual drug treatments **(revised Supplemental Figure 3E)**. This discrepancy may be attributed to the longer incubation time required for low MOI infections, which could impact host gene expression, as noted by Reviewer #3. We have addressed this point in the revised discussion **(lines 486-490)**.

3) The authors report that influenza and coronavirus replication are more significantly inhibited that other picornaviruses by the drug treatments used. While this is of interest the authors don't acknowledge that these viruses use cap dependent translation, similar to the first ORF in the reporter used raising concerns about the robustness of the assay reported in 6B.

We apologize for any confusion regarding the differential effects of kinase inhibitors on coronaviruses, influenza viruses, and picornaviruses. Specifically, SRPKIN-1 inhibited human coronaviruses, whose nucleocapsid protein (NP) contains an SR-rich motif phosphorylated by SRPK1. This phosphorylation masks the RNA-binding domain of NP, thereby disrupting ribonucleoprotein (RNP) assembly. In contrast, TG003 suppressed human influenza A virus replication by targeting the splicing of viral mRNAs, a critical process for efficient viral protein synthesis. To better clarify these mechanisms, we moved these data to the revised **Supplemental Figure 3D** and elaborated on the underlying mechanisms in **lines 385-390**.

Regarding the reviewer’s point about the role of phospho-SR proteins in cap-dependent translation, this is indeed an important question. Hypophosphorylated SR proteins are known to interact with the mRNA export receptor NXF1 to facilitate mRNA export and activate cap-dependent translation, suggesting that the dephosphorylation of SR proteins may play a role in this process (lines 423-427). However, as mentioned by Reviewer #3, it remains unclear whether other picornavirus IRES types, such as those in HCV or CrPV, which are less dependent on ITAFs, are also regulated by phospho-SR proteins. This question warrants further investigation, as the distinctions between cap- and IRES-dependent translation remain an area of our ongoing research **(lines 502-506)**.

We have revised the manuscript to specifically emphasize the regulation of phospho-SR proteins in picornavirus type I IRES-dependent translation **(lines 381-384)**. Moreover, to strengthen our current findings, we performed a mono-luciferase assay using RNA transcripts to demonstrate the role of phospho-SR proteins in EV-A71 IRES-dependent translation **(revised Figure 7F)**.

Although our study provides strong evidence for the importance of phospho-SR proteins in regulating picornavirus IRES translation, a detailed investigation into their distinct roles in cap- and IRES-dependent translation mechanisms is beyond the scope of the current manuscript. These findings are part of our ongoing studies, and we aim to publish this work in a future report. We appreciate the reviewer’s suggestion, which has guided us to improve our discussion and highlight the specific contributions of this study.

**Minor Issues: Editorial and Data Presentation**

**Reviewer #2:**

1) In the introduction, the authors move between writing about virus replication in general versus picornavirus replication more specifically, this gets confusing. For example, the first paragraph describes +sense SS RNA virus replication, then jumps to general replication in the second paragraph asserting that translation is essential in the eclipse phase on line 88 without any real connectivity, this should be rewritten to make any point clearer.

We appreciate the reviewer’s feedback regarding the clarity of the introduction and have revised the text to address these concerns **(lines 76-97)**. Specifically, we have emphasized the focus on positive-sense (+) single-stranded RNA (ssRNA) viruses, ensuring a clear distinction between general virus replication and the replication cycle of this virus group. The revised text highlights conserved viral mechanisms, such as the RNA-dependent RNA polymerase (RdRp), as an example of a successful antiviral target. Building on this, we have expanded the discussion to emphasize the critical role of virus-host interactions in replication.

This shift underscores the importance of exploring conserved mechanisms that rely on virus-host dynamics for broad-spectrum antiviral development. Additionally, we have improved the flow by logically transitioning from the general replication cycle to the specific regulation of translation and replication in (+) ssRNA viruses, including the importance of the translation-replication switch. These revisions aim to provide a more cohesive and focused introduction, directly addressing the reviewer’s concerns.

2) I was very surprised that the authors omitted any reference to previously published work on SR proteins and enterovirus translation/replication given the clear relevance to this report. The excellent work from PMID: 17183366, PMID: 21779168, PMID: 23255796 describing the role for SRp20 in poliovirus translation/replication should be acknowledged and discussed- for example could the inhibitors tested impact SRp20 function?

We apologize for the oversight in our initial manuscript, where we relied primarily on the review by Flather and Semler (PMID: 26150805) to summarize the role of SRSF3. In the revised manuscript, we have expanded the discussion to include a more comprehensive review of the role of SRSF3 in poliovirus infection, specifically addressing its 2A protease-dependent cytoplasmic relocalization and its function in IRES-dependent translation **(lines 99-102 and 116-122; cited as references 11, 15, and 16)**. Additionally, we performed siRNA experiments to investigate the role of SRSF3 in EV-A71 infection. However, we observed only a moderate, non-significant reduction in viral replication **(now shown in revised Figure 3F)**. Furthermore, we did not detect significant upregulation of SRSF3 phosphorylation upon EV-A71 infection **(revised Figures 4B, and 7A)**. These findings suggest that different SR proteins may regulate viral infection through distinct mechanisms, as discussed in the revised Discussion section **(lines 497-499 and 502-514)**.

We appreciate the reviewer highlighting the relevance of prior studies, and we have now incorporated these important references and expanded the discussion to address the potential functional impact of SR protein inhibitors on SRp20/SRSF3.

**Reviewer #3:**

The manuscript by Lee et al, entitled “Subversion of Phosphorylated SR Proteins by Enterovirus A71 in IRES-Dependent Translation Revealed by RNA-Interactome Analysis” describes a commonly used approach to in vivo label RNA and crosslink proteins bound to the RNA for mass spec identification. The group identified 183 proteins interacting in early during EV71 infection, while 181 were identified late in infection. Only 44 were identified both early and late. While this study is in principle very important and useful to the field, there were many controls that were not included in both the mass spec analysis such as mock infected cells. This is particularly important as it seems like their inhibitor of cellular mRNA transcription was not effective so the 4sU labeled RNAs contained both host and viral RNA thus necessitating a control with only host mRNA. This would allow them to identify RNA binding proteins that were enriched in the virally infected cells. They focused on SR proteins but in attempting to validate the roles of the proteins in viral infection they did not do any knockdown experiments. There were only a few SR proteins so this should have been feasible even if they needed to knock them down in combinations. Rather they focused on performing a number of suggestive experiments that were not compelling largely due to the lack of important controls (see comments below). However, Figure 3D-E showing relocalization of the SR proteins was convincing. Overall, the enthusiasm for this manuscript is limited as many of their conclusions are not justified by the data presented.

We thank the reviewer for their detailed evaluation and constructive feedback, which have greatly improved the clarity and rigor of our study. In response to the concerns raised, we have substantially revised the manuscript to address key issues, including clarifying experimental details, incorporating additional controls where feasible, and expanding the discussion to acknowledge limitations and contextualize our findings.

While we did not include mock-infected controls in the mass spectrometry analysis, we have carefully addressed this limitation in the revised manuscript. Additional figures and supplemental data now provide clarity on the contributions of host and viral RNA, with detailed discussions on transcription inhibitor efficacy and RNA labeling. In the revised manuscript, we have also conducted knockdown experiments for several SR proteins and validated their functional roles through complementary approaches, including luciferase assays, protein expression analysis, and inhibitor studies.

To ensure transparency and reproducibility, we have expanded the Materials and Methods section and figure legends to provide comprehensive details on replicates, MOI, drug concentrations, and experimental conditions. We believe these revisions strengthen the conclusions of our study and enhance its contribution to the field. Please find detailed explanations and data to support our claims in the point-by-point responses.

**Major Issues: Key Experiments Required for Acceptance**

**Reviewer #3:**

1.	It appears from figure 1C and potentially the lack of effect on viral growth that the alpha-amanitin was not effective at inhibiting host transcription after 6 hours (-2 to 4 hpi). At later times in infection (8 hpi) the virus inhibits host transcription and nuclear export, which probably explains the drop in some mRNAs but not others dependent on their half-life. It seems to this reviewer that the point of using the Pol II inhibitor is to only label the viral RNAs however Figure 1C (lane 11) shows this clearly didn’t work.

We appreciate the reviewer’s insightful comments regarding the inhibition efficiency of α-amanitin and its implications for our experimental design. To determine optimal transcriptional inhibition conditions in human rhabdomyosarcoma (RD) cells, we tested varying concentrations of transcriptional inhibitors: actinomycin D (ActD), α-amanitin, and 5,6-dichlorobenzimidazole 1-β-D-ribofuranoside (DRB), by measuring mRNA half-life for several transcripts, including *ACTB*, *VIM*, *GAPDH*, and the stable transcript *MED16* (PMID: 23303783) **(Supplemental Figure 1)**. These experiments demonstrated that ActD provided the most robust inhibition, followed by α-amanitin and DRB, with all inhibitors reducing mRNA levels by more than 50% after 2 hours of treatment. A representative RT-qPCR experiment using 4 μM ActD, 100 μM DRB, and 2 μM α-amanitin is now included in **revised Supplemental Figure 1 and described in lines 152-156**.

Using these findings, we designed a 2-hour pre-treatment in subsequent experiments to inhibit host transcription. However, while mRNA levels were substantially reduced, residual transcription activity was sufficient to label host transcripts, contributing noise to the viral RNA-protein interactome. To address this, we have avoided using the term “viral RNP” throughout the manuscript and explicitly acknowledged the presence of host-derived transcripts **(lines 166-173, and lines 223-225)**.

We believe these clarifications and additional data provide a more transparent and comprehensive understanding of the limitations and rationale underlying our experimental approach.

2.	In Figure 1 the authors use RT-PCR (presumably reverse transcriptase followed by non-quantitative regular PCR), which is not quantitative and can only confirm presence or absence of the RNAs. Thus, lines 158-160 “RT-PCR analysis confirmed a comparable level of viral and host transcripts being pulled down (Figure 1D)” should be revised to say both viral and host transcripts were pulled down.

We apologize for the use of non-quantitative agarose gel electrophoresis to compare the levels of pulled-down RNA in our initial submission. To address this, we now provide a quantitative analysis of three technical repeats from one representative experiment **(revised Figure 1E)**. The revised data show that α-amanitin treatment improved the pulldown efficiency of viral RNA at 4 hours post-infection (hpi) from 12.2% to 23.9%, which is comparable to the levels of viral transcripts observed at 8 hpi in the untreated group (24.9%). Additionally, the pulldown efficiency of viral transcripts was similar to that of highly abundant host housekeeping genes **(lines 166-172, and lines 223-225)**.

Although no additional data is included in the revision, we also tested the pulldown efficiency using a higher multiplicity of infection (MOI 40) and repeated the experiments several times. While the absolute pulldown efficiency varied between replicates, a consistent trend was observed across all experiments.

These results further highlight the interference of host transcripts in our analysis. As described in response to Question 1, we have explicitly acknowledged this interference and its implications.

3.	In the pull-down of RNPs the authors used non-4sU as their control, but a better control to ensure they were not identifying proteins bound to cellular mRNAs would be to use 4sU labelled uninfected cells. Since this would allow them to look for enhancement of the RNA binding proteins over background (uninfected cells).

We appreciate the reviewer’s insightful suggestion regarding the use of 4sU-labeled uninfected cells as a control. While this approach could help identify proteins bound exclusively to cellular mRNAs, we opted to use non-4sU-labeled samples at each time point as our control to focus on 4sU-specific binding during infection. This strategy allowed us to perform quantitative proteomic analysis based on the differences in average normalized spectral counts between 4sU and non-4sU groups at specific time points, ensuring that observed changes reflected the dynamics of infection rather than variations in protein expression or abundance. Since "background" protein levels may vary significantly across time points due to infection-driven changes, performing cross-time-point quantitative analysis would not yield reliable insights. Instead, we employed Venn diagram analysis to compare RNPs captured at 4 and 8 hpi, identifying proteins specifically enriched at each stage of infection.

We acknowledge that, in the absence of a mock-infected control, many identified proteins may still associate with host transcripts, making it challenging to distinguish between host- and viral-derived RNA interactions. However, as described in the manuscript, binding to host mRNA does not preclude the possibility that these proteins also bind to viral RNA **(lines 243-246).** Such interactions are not mutually exclusive, and their presence in the dataset remains relevant to understanding RNA-protein dynamics during infection. Nonetheless, we recognize the limitations of this approach and emphasize that further validation is required to confirm the viral RNA association of the identified RNPs. These clarifications have been included in the revised manuscript **(lines 439-443)**.

4.	The MS analysis of RNP complexes appears to have been performed only once. Given how non-specific biotin-strep interactions are and the amount of background this can produce, it would be important to know how reproducible the 227 and 225 proteins are that they found. Also, consistent with them using the wrong control (see previous comment) that doesn’t control for RNPs bound to host RNAs the number of proteins they identified is rather large.

We appreciate the reviewer’s concerns regarding the reproducibility of our MS analysis and the large number of proteins identified. First, we would like to clarify that the RNP complexes were not isolated using biotin-streptavidin interactions. Biotin-streptavidin was used solely to confirm that viral transcripts were successfully labeled, as shown in the **revised Figures 1D and 1E**. For RNP isolation, the thiol groups on 4sU-labeled RNAs were photoactivated using 365 nm UV light to crosslink with nearby proteins. Given the potential impact of crosslinked proteins on the viral sequence and the lack of virus-specific biotinylated probes when the assay was initiated, we employed Oligo(dT) magnetic beads to purify poly(A)-containing transcripts for RNP analysis **(lines 622-626)**. The limitations of using Oligo(dT) for RNP isolation have been extensively discussed in our responses to Questions 1-3.

Due to equipment limitations, the MS analysis was performed only once. However, we conducted technical replicates to ensure the reliability of the proteomic results **(lines 657-664)**. Before proceeding with MS, we repeated RNP pulldown experiments using different MOIs and tested various amounts of crosslinked lysates with Oligo(dT) beads. Only after we consistently differentiated the 4-hour and 8-hour RNP profiles did we scale up the pulldown for MS analysis.

While the MS experiment was conducted once, we validated the key interactions using RNA immunoprecipitation (RNA-IP) and RNA fluorescence in situ hybridization (FISH). Additionally, we have initiated further studies to characterize the RNA association of stress granule, ER, and mitochondrial proteins and their roles in viral replication. These ongoing and accumulated validations strengthen our confidence in the accuracy and relevance of the MS results.

5.	Figure 3A shows that a broad SR antibody pulls down EV-A71 RNA and ACTB mRNA, however, the authors do not include a negative control of an mRNA that is not present in the 16H3 pull-down, the concern is more that it is not specific rather than that EV-A71 can still be pulled down even when cellular mRNAs are present. It appears that even their IgG1 control pulls down EV-A71 RNA granted to a lesser extent but could this just be a reflection of how many copies of viral RNA are present in the cells?

We appreciate the reviewer’s comments and apologize for the inadequacy of our initial conclusions based on non-quantitative RNA-IP results. We also thank the reviewer for highlighting the potential non-specific association of abundant RNAs in the RNA-IP assay. To address this concern, we conducted quantitative PCR (qPCR) to evaluate the RNA-IP efficiency by normalizing the IP signals to those of the input samples **(revised Figure 3D)**. In the infection group, viral transcripts were clearly detected and showed IP efficiency comparable to that of ACTB mRNA. This analysis confirms the specific association of viral RNA with SR proteins in the context of infection **(lines 243–246)**.

Furthermore, to demonstrate the functional importance of SR proteins identified using the pan-SR antibody, we performed siRNA-mediated knockdown experiments **(revised Figures 3E and 3F)**. The results indicate that knockdown of SRSF4, SRSF5, SRSF6, and SRSF7 significantly impaired EV-A71 replication **(lines: 246-254)**. By integrating the findings from the RNA-IP experiments, the SR protein knockdown experiments **(revised Figure 3)**, and the RNA FISH data **(revised Figures 4F and 4G)**, we provide a more comprehensive validation of the role of specific SR proteins in supporting EV-A71 replication. We believe these additional analyses adequately address the reviewer’s concerns and clarify the importance of SR proteins in this context.

6.	Figure 3B, the authors make the claim “however, alterations in their phosphorylation status, particularly an increase at 4 hpi, were evident”. This reviewer does not find the increase in SR protein phosphorylation obvious. In fact, given that the methods specify HRP, which is not quantitative relative amounts cannot be quantified.

We appreciate the reviewer’s concerns, which were also raised by Reviewer 2 (point 2). We acknowledge that the use of HRP-based detection is not inherently quantitative and that clearer evidence is necessary to support our claims. To address this important issue, we repeated all experiments comparing the phosphorylation signals and collected digital signals using a CCD camera for subsequent quantitative analysis with commercial software **(revised Figures 4A–4C, 7A–7B, Supplemental Figures 2 and 3C; Materials and Methods, lines 650–655)**. Based on these quantitative results, we have specified the fold changes in phosphorylation levels at different time points in the revised manuscript **(lines 260–270)**.

We believe these revisions provide more robust and quantitative evidence to support our observations regarding the changes in SR protein phosphorylation during EV-A71 infection.

7.	Figure 5A, it is surprising that the polysomes in the mock are so low. One might guess that the cells were not fed, that they may be over confluent, or that cycloheximide or another elongation inhibitor was not added. Typically, mock cells should have robust polysomes but as the RNA gel shows in Fig 5B most of the ribosomes are in the 40S to 80S fractions. Under these conditions it is impossible to determine whether SR proteins would be associated with the polysome fraction since there are essentially no polysomes. Given the improved but still low polysomes in 5E, it may be a combination of issues. The conclusion: “However, following EV-A71 infection, phospho-SR proteins and SR proteins specifically accumulated in fractions containing 40S and 80S monosomes” does not match the data as there is clearly a robust amount of SR proteins in the free mRNP fractions (1-3). Is there any reason why in the mock that ACTB would not be detected by RT-PCR in fractions 8 and 9? Was this only performed once or was this reproducibly observed. It seems unexpected.

We appreciate the reviewer’s observations and concerns regarding the data and interpretations in Figure 5 of the original submission. Upon review, we acknowledge inconsistencies in the original submission, which arose from combining data contributed by different co-authors. While the data were originally confirmed through more than three experiments to ensure reproducibility, we have repeated key experiments to address the reviewer’s specific concerns. In the revised manuscript, we repeated the polysome profiling of EV-A71-infected cells at 4 and 8 hpi **(revised Figure 6A)**. These experiments were performed using RD cells at 90-95% confluence, cultured with 10% FBS, and treated with cycloheximide (CHX) at a final concentration of 100 μg/mL for 15 minutes before lysate preparation. To preserve polysomes, cells were washed with CHX-containing buffer and kept at 4°C during sample preparation. These methodological details are now included in the revised Materials and Methods section **(lines 782–787)**.

In the **revised Figure 6A**, we observed more *28S* and *18S* rRNA in the heavier fractions in the mock group, consistent with robust polysome formation. However, EV-A71 infection caused a shift of *28S/18S* rRNA toward lighter fractions, indicating disrupted polysome assembly. This shift was corroborated by RT-PCR, which showed that *ACTB* transcripts also shifted toward lighter fractions during infection. In the mock group, *ACTB* transcripts were consistently detected in fractions 6–15 across experiments. Therefore, we believe the absence of detectable *ACTB* in fractions 8 and 9 in the mock group in the original Figure 5A was due to sample loss or poor RNA recovery during preparation.

Additionally, we rephrased the manuscript to acknowledge that phospho-SR proteins predominantly distribute in the free mRNP fractions **(revised lines 331–341)**. To improve clarity and consistency, we also reorganized the data: RNA distribution profiles are now presented in **revised Figure 6A**, while western blot results have been moved to **revised Figures 6D and 6E.** These revisions address the reviewer’s concerns about the data presentation and interpretation.

8.	The conclusion: “These results demonstrate the presence of phospho-SR proteins in the translation initiation complex upon EV-A71 infection, suggesting that EV-A71 may redirect nuclear phospho-SR proteins to regulate translation in the early stages of infection.” is not justified as the Mock and EV-A71 CHX treated lysates were not shown. While there is a small amount of polysomes and it is more substantial than those shown in fig 5B, a control showing the CHX blot is needed to compare to the EDTA and Puro blots. Importantly, we cannot compare the westerns in figure 5C to those in Fig. 5F since these appear to be different gradients and there is a slight shift in the fractions.

We apologize for the inconsistencies between Figures 5C and 5F in the original submission, as previously discussed in our response to Point 7. To address this issue, we have included western blots for the mock/CHX and EV-A71/CHX conditions in the **revised Figures 6D and 6E**, corresponding to the polysome profiles shown in **revised Figure 6B**.

Based on the updated results, phospho-SR proteins were detectable in the polysome fractions under CHX treatment. Importantly, these signals disappeared following puromycin or EDTA treatment, confirming their association with active translation complexes. To ensure clarity and accuracy, we have revised the text to describe that while phospho-SR proteins predominantly distribute in the free mRNP fractions, they are also detected in fractions containing the 40S, 80S, and polysomes under EV-A71 infection **(lines 331-341)**. These revisions provide a more accurate representation of our findings and address the reviewer’s concerns about the conclusions drawn from the original data.

9.	Figure 6A, it is unclear to this reviewer which SR protein is phosphorylated. Generally, proteins migrate slower when phosphorylated in 6A there is a band around 70KD that maybe correlates to a band around 50KD, while another band around 185 KD moves to under 180KD in the pSR blot. Could these blots be mis-labeled or are the different SR proteins showing up on different blots? Also, why are there only 3 SR proteins on the lower SR western?

We apologize for the unclear labeling and the resulting confusion in the original submission. Both clone 16H3 (α-SR) and 1H4 (α-pSR) antibodies are broad-reacting antibodies, but they are raised against different SR proteins and target distinct epitopes. Consequently, the major bands detected by one antibody may not correspond to bands detected by the other, leading to apparent discrepancies in the western blot results. To address this issue and clarify the effect of the kinase inhibitor on each SR protein, as also suggested by Reviewer 2, we performed a literature review and siRNA knockdown experiments to identify the major SR proteins detected by each antibody **(lines 236-241; revised references 41-45)**. In the **revised Figures 3–7**, we have annotated each identified protein in the western blots to enhance clarity.

Furthermore, as mentioned in response to Question 6, we repeated the experiments using a CCD camera to collect digital signals for quantitative analysis. These results are now presented in **revised Figures 7A and 7B, as well as Supplemental Figure 3C**, with clear labeling and quantification to ensure the reliability and interpretability of our findings.

10.	Figure 6B is missing a positive control whereby an IRES is used that is not affected by SR proteins and shows that these inhibitors have no effect on general translation. One possible IRES could be the CrPV IGR IRES as this is a minimal IRES not known to require ITAFs.

We greatly appreciate the reviewer's insightful suggestion. As also highlighted by Reviewer 2, it remains an open question whether cap-dependent translation and other picornavirus IRES types, such as those in HCV or CrPV—which are less dependent on ITAFs—are similarly regulated by phospho-SR proteins. This intriguing possibility warrants further investigation, as the distinctions between cap- and IRES-dependent translation represent an important focus of our ongoing research. To address this point, we expanded our discussion on the potential role of phospho-SR proteins in cap-dependent translation and their broader regulatory implications **(lines 426-430, 502-507)**. Additionally, to strengthen our current findings, we conducted a mono-luciferase assay using RNA transcripts to confirm the role of phospho-SR proteins in EV-A71 IRES-dependent translation **(revised Figure 7F)**. The manuscript has been revised to specifically emphasize the regulatory role of phospho-SR proteins in picornavirus type I IRES-dependent translation **(lines 381-384)**.

11.	Given that IRES activity is reported as a ratio, this assumes that Rluc is constant across all conditions and all the changes are due to Fluc values changing. Best practices are to present both Fluc and Rluc raw values for each replicate in a supplementary table.

We thank the Reviewer for their insightful comment, which improves the clarity of our dual-luciferase assay results. The raw values of Renilla and Firefly luciferase activities are now presented in **Supplemental Figure 4 (lines 370-377)**.

12.	Also figure 6C lacks a good control that shows that these inhibitors do not affect translation of a virus that does not rely on SR proteins, one that doesn’t have an IRES such as HSV or VSV. It would also be nice to see that 35S methionine incorporation was not affected by either of these inhibitors. Any negative effect on the translation machinery could non-specifically impact viral IRES translation or viral production.

As highlighted in our response to Question 10, the distinctions between cap-dependent and IRES-dependent translation remain a critical focus of our ongoing research. We have revised the manuscript to specifically emphasize the regulatory role of phospho-SR proteins in picornavirus type I IRES-dependent translation **(lines 381-384)**. Regarding cap-dependent translation, as described in our response to Question 11, we observed that it was not affected, and in some cases, appeared to be enhanced **(Supplemental Figure 4)**. To further specify the role of phospho-SR proteins in EV-A71 IRES activity, we performed a mono-luciferase assay using RNA transcripts, which confirmed the involvement of phospho-SR proteins in EV-A71 IRES-dependent translation **(revised Figure 7F, lines 377-381)**. Although we did not conduct a 35S methionine incorporation assay, we assessed the expression levels of EV-A71 viral proteins 3CD and VP0 upon treatment with kinase inhibitors and performed quantitative analysis **(revised Figures 7A and 7B)**. These results were consistent with the observed reduction in viral protein expression and the subsequent decrease in viral titers **(revised Figure 7C, lines 356-361)**.

We sincerely thank the reviewer for their comments, which helped us refine and strengthen the conclusions presented in our study.

13.	Given that the kinase inhibitors, SRSRPKIN-1 and TG003, reduced viral plaques for a range of RNA viruses, it is important for the authors to make sure that these kinase inhibitors are not reducing fitness for viral amplification by affecting the cell fitness or have off target effects (as many kinase inhibitors are known to have). The most robust method would be to show that an unrelated DNA virus was not affected by these inhibitors. Alternatively, a cell viability assay could be performed.

We appreciate the reviewer’s suggestion to address the potential off-target effects and cytotoxicity of the kinase inhibitors SRSRPKIN-1 and TG003. Indeed, we tested the cytotoxicity of each drug, both individually and in combination, to ensure that the observed antiviral effects were not due to reduced cell fitness. We apologize for not including this data in the original submission. This information is now described in the revised manuscript **(lines 350-353)** and presented in **Supplemental Figure 3A and 3B**.

Furthermore, the potential impacts of these inhibitors on host gene expression have been discussed in the revised manuscript **(lines 486-490)**. While we acknowledge that testing an unrelated DNA virus would provide additional robustness, the cytotoxicity assay results, along with our discussion on potential off-target effects, offer critical insights into the specificity of these inhibitors.

14.	Overall, it seemed to this reviewer that most experiments were performed only once. In general, there was no reporting of technical or biological replicates, MOI, etc. Figure legends were overly brief, the methods section was also not sufficient to understand how most of the experiments were performed.

We thank the Reviewer for their thorough assessment and suggestions, which helped us improve the clarity and robustness of our manuscript. We understand the importance of providing detailed information regarding experimental replicates, MOI, drug concentrations, and methodology. To address these concerns, we have revised the **Materials and Methods** section and expanded the **figure legends** to ensure transparency and reproducibility. Specifically:

**Replicates:** We have clarified in the revised manuscript that all experiments were performed with at least three independent biological replicates unless otherwise noted. Additionally, technical replicates (e.g., for qPCR and luciferase assays) were conducted and are indicated in the legends.

**MOI and Drug Concentrations:** The MOI used in each experiment and the concentrations of inhibitors or drugs are now explicitly stated in both the Materials and Methods section and figure legends.

**Experimental Details:** Extensive rephrasing has been applied to ensure that the **Materials and Methods** section provides sufficient details about how each experiment was conducted, including the protocols for infection, drug treatment, metabolic labeling, and luciferase assays.

**Efforts and Repetition:** As indicated in responses to prior points, we have re-performed key experiments to address the Reviewers’ concerns and provide additional controls **(e.g., Supplemental Figures 4 and 7A)**. We hope this demonstrates the significant efforts we have made to ensure robust and reliable results.

We have also added raw data (e.g., luciferase values and PCR results) and supplementary figures to provide further clarity. With these revisions, we believe the manuscript now addresses all concerns raised about replicates, experimental details, and clarity in figure legends.

**Minor Issues: Editorial and Data Presentation**

**Reviewer #3:**

1.	Figure legends are not adequate: Figure 1B – how was the RNA and virus levels measured? What concentration of the drugs were used? 1C-D) It is unclear when the authors are using reverse transcription and PCR or real-time qPCR. This needs to be made clear. Presumably since they showed bands on a gel this is regular PCR, which brings up the concern that their conclusions are not supported by the data. Figure 3A what does the arrow and asterisk indicate? Also, what are the expected sizes of the SR proteins? Could the SR proteins be labelled? Also, how many hpi was the pull-down performed? What MOI was used? How many cycles was the PCR reaction run for?

We thank the Reviewer for their constructive comments regarding the figure legends and experimental details. In the revised manuscript, we have made the following changes:

**Figure 1B:** The methods for RNA and virus quantification are now explicitly stated in the figure legend. Viral titers were measured using plaque assays, and RNA levels were quantified by reverse transcription-quantitative PCR (RT-qPCR). Drug concentrations (e.g., 2 μM α-amanitin, 5 μM ActD, and 100 μM DRB) are now included in the legend.

**Figure 1C-D:** We clarified when reverse transcription followed by either PCR or RT-qPCR was performed. Bands shown on gels in revised Figure 1D are from conventional PCR, and the corresponding primer sets and cycle numbers are now detailed in the **Materials and Methods section (lines 599-610)**.

**Figure 3A:** The arrow and asterisk indicate the immunoglobulin heavy chain (IgH) and light chain (IgL), respectively. The SR proteins corresponding to the major bands detected by broad-acting antibodies are explicitly labeled in both the figure and its legend.

Additionally, details such as the MOI used for infection (MOI 10), the time points of pull-down (e.g., 4 or 8 hpi), and PCR cycle conditions have been clarified in both the figure legend and the Materials and Methods section. We hope these clarifications adequately address the Reviewer’s concerns and improve the overall clarity and rigor of the manuscript.

2.	Figure 2B-E, it’s difficult to read the protein names due to how busy the figure is with the dark colors and black type.

To address the concern about readability in Figure 2B-E, we have included the raw data of 4- and 8-hour RNPs as Rebuttal Figure 1. This figure was generated using Cytoscape (URL: https://cytoscape.org/), and we have adjusted the contrast to improve clarity. While we suspect the resolution may have been affected by PDF compression during submission, we will ensure the resolution and clarity are optimized in the final submission to make the protein names more legible.

**Rebuttal Figure 1.** Raw data of 4- and 8-hour RNPs used for constructing Figure 2. Generated using Cytoscape.

3.	Typos: Figure 1D Vrial should be Viral, Fig 3C tranlocation should be translocation. line 98 tuns should be tunes.

We sincerely apologize for the typographical errors in the original submission. These have been corrected as follows:

In revised **Figure 1G** (formerly Figure 1D), 'Vrial' has been replaced with 'vRNA' to represent viral RNA.

In revised **Figure 4E** (formerly Figure 3C), 'tranlocation' has been corrected to 'translocation.'

In revised **line 104** (formerly line 98), 'tuns' has been corrected to 'fine-tunes.'

These corrections are reflected in the revised manuscript and figures.
