# Supplementary material for: Subversion of phosphorylated SR proteins by enterovirus A71 in IRES-dependent translation revealed by RNA-interactome analysis
Source: PLoS Pathog. 2025 Jun 16;21(6):e1013242. doi: 10.1371/journal.ppat.1013242 (PMC12193706; doi:10.1371/journal.ppat.1013242)
Supplement: Attachment — Submitted filename: Submission Response to Reviewers.docx [file ppat.1013242.s010.docx]

We thank the editor and reviewers for their constructive feedback and thoughtful comments. A summary of the major revisions is provided in the cover letter. Below, we provide a detailed point-by-point response to each comment.

**Reviewer 1**

The revised manuscript by Lee et al., has been greatly improved by adding siRNA knockdown of individual SR proteins and performing quantitative analysis of the interactome. Overall, the quality of the figures has been improved. In addition, authors incorporated a detailed description of methods and results, and a more complete discussion on the SR proteins in viral RNA translation and virus multiplication. In my opinion, the conclusions are novel and supported by solid results, despite future work is needed to understand the mechanism of action of all these proteins.

We sincerely thank Reviewer #1 for the encouraging and thoughtful feedback. We are grateful that the reviewer recognizes the improvements in the revised manuscript, including the addition of siRNA knockdown experiments, quantitative analyses, and the expanded discussion. We appreciate the reviewer’s positive assessment of our conclusions and hope that our study will provide a valuable foundation for future research on the roles of SR proteins in viral RNA translation and replication.

**Reviewer 2**

Lee at al. have submitted an updated version of their paper reporting the RBP interactome of EV71 at different timepoints after infection. The paper is much improved in writing and depth of discussion and details on experiments included.

The paper is still ambitious and uses cutting edge techniques for determining viral RNA interactome at different viral replication stages. The results are potentially novel and could significantly add to the field.

The study uses metabolic labelling of newly synthesized RNA during infection after inhibition of cellular transcription to enrich for viral RNA cross linking to interacting proteins. However, the experimental set up does not distinguish between protein hits that bind to viral RNA or residual host mRNA.

The authors follow up on the SR family of proteins identified and perform experiments to test their impact on EV71 replication.

We sincerely thank Reviewer #2 for the positive feedback on the revised manuscript and for recognizing the improvements in both writing and experimental clarity. We appreciate the reviewer’s recognition of the novelty and potential impact of our study, as well as the acknowledgment of the ambitious scope and technical strengths of our approach.

We also appreciate the reviewer’s continued concerns regarding the specificity of the RNA-protein interactome and the potential contributions of host mRNA. We have now addressed this point directly in the revised manuscript and in our point-by-point responses below, including additional experiments and discussion to clarify the limitations and strengths of our approach. Please find our detailed responses following.

Major comments:

1. A major issue affecting the robustness of the paper’s conclusions is still quantification of protein and phospho protein levels. This is particularly affecting conclusions in figure 4A where no loading control is included in A or B, while tubulin is shown in panel C (4hrs) but is cleaved at 8 hours. If Tubulin is not a suitable loading control due to its cleavage, why not use a different loading control. Although the authors have changed their analysis procedure for quantifying gels, the small changes of 10-20 % are difficult to interpret on the gels shown and the relevance of such small changes is not clear. It looks like all signals in the gels change in a similar direction whether they are labelled as SR proteins or are non specific proteins.

We thank the reviewer for this insightful comment regarding the quantification and interpretation of protein and phospho-protein levels. We also apologize for the inadequate organization of Figure 4A, 4B, and 4C in the previous submission. As the data for 4 hpi and 8 hpi were derived from the same blotting experiment, we have now reassembled all relevant panels into a single, revised Figure 4A to ensure transparency and facilitate direct comparison across conditions. This updated layout clarifies that the different bands originate from the same experimental set.

We agree with the reviewer that tubulin is not a suitable loading control due to its cleavage at 8 hpi (lines 299-300). Instead, we assessed GAPDH expression, which remained consistent across all time points without any detectable truncation. GAPDH was therefore used as a reliable internal control for normalization. To address concerns regarding protein loading, we have re-quantified all bands using GAPDH for normalization, and the results are presented in the revised Figure 4B (lines 288-297).

To further support our conclusion that the observed changes are not simply due to a global up- or downregulation of total protein levels, we provide Supplemental Figure 3A, which presents densitometric profiles from mock- and EV-A71-infected samples. These profiles highlight specific SR proteins (e.g., SRSF4, SRSF5, SRSF6 and SRSF3) with distinct changes in intensity and migration patterns following infection (lines 292-295). Importantly, these alterations are not observed uniformly across all bands, including those potentially detected non-specifically by the pan-antibody. This suggests that the observed changes reflect selective regulation rather than general alterations in total protein abundance.

2. Of more concern, and I may be missing something here, is Figure 7A and B. The authors use drug treatments to inhibit phosphorylation of SR proteins and conclude that the phosphorylation state impacts virus replication and IRES activity. But in 7 A and B the decrease in the phosphorylated SR protein signal (IB: anti pSR) appears to correlate in both the gel and the quantification panel with the decrease in the total protein (IB: anti-SR) suggesting that the ratio of phospho to non phospho SR proteins (which is not shown but should be) does not change with the drug treatment. If this is the case then the conclusions and discussions on this aspect of the paper are not substantiated.

We thank the reviewer for the valuable comment and the opportunity to clarify our data interpretation. As correctly noted, both total SR and phospho-SR protein levels were reduced following combined treatment with TG003 and SRPKIN-1 (original Figure 7A). One possible explanation for this decrease is reduced protein stability or degradation after dephosphorylation, similar to what we observed during the later stages of infection (see Figure 4A). Another possibility is that the pan-SR antibody used in our study may also recognize certain phosphorylated or partially phosphorylated forms of SR proteins, as SR proteins can exist in multiple phosphorylation states (e.g., hyperphosphorylated, hypophosphorylated). Therefore, a reduction in the phosphorylation state could lead to a partial decrease in the total signal detected by the pan-SR antibody, given that phosphorylated forms constitute a substantial portion of the total SR protein pool. To further support this possibility, we conducted additional experiments using two other SR protein kinase inhibitors (Compound T and Compound CL) as part of our ongoing research (see Rebuttal Figure 1). These treatments consistently resulted in a more pronounced reduction in phospho-SR protein levels compared to total SR protein levels, approximately a two-fold greater decrease suggesting that the observed changes may reflect a specific loss of phosphorylation rather than general protein degradation.

We have now replaced the original Figure 7A with this new dataset and updated the quantification accordingly. In the revised Figure 7B, the relative levels of total SR and phospho-SR proteins under different drug treatments are presented side by side, normalized to GAPDH, to more clearly show the differential impact on phosphorylation. The text in the main manuscript and figure legend has been revised to reflect this update (lines 393-400, 1344-1349).

**Rebuttal Figure 1. Alteration of SR and phospho-SR protein levels in EV-A71-infected RD cells following treatment with different SR protein kinase inhibitors.** Left panel: Representative western blot showing SR and phospho-SR protein levels in RD cells infected with EV-A71 (MOI = 20) and treated with TG003, SRPKIN-1, or two additional experimental kinase inhibitors (Compound T and Compound CL). Right panel: Quantification of total SR and phospho-SR protein levels from three independent experiments, normalized to GAPDH. Notably, phospho-SR proteins consistently exhibited a more pronounced reduction than total SR proteins across treatments, supporting a specific decrease in phosphorylation levels.

3. A second issue is still the lack of a mock infected control to try and determine which hits are due to interaction with viral or host RNA that is still present due to purification by oligodT beads.

We appreciate the reviewer’s continued attention to the specificity of our RNA-protein pulldown strategy and the concern regarding the absence of a mock-infected control. We acknowledge that including a mock-infected control could help distinguish proteins bound to host mRNAs from those specifically associated with viral RNAs. However, EV-A71-induced host shutoff significantly alters global transcription and translation, making direct comparisons to mock-infected samples potentially misleading due to differences in total RNA and protein composition. This limitation has been noted in previous studies of virus-host interactomes (e.g., Iselin et al., Trends Biochem Sci, 2022, PMID: 34509361). To mitigate this, we conducted proteomic comparisons between 4sU-labeled and non-labeled (-4sU) samples collected under the same infection conditions, ensuring that both groups experienced equivalent host shutoff dynamics and global expression profiles. This design allows us to identify proteins specifically enriched in the 4sU-labeled pulldown within an infection-matched background. These limitations are now acknowledged in the revised manuscript (lines 208-211).

As requested by Reviewer #3 (Major Point 3), and to confirm the specificity of the oligo(dT) pulldown, we performed RT-qPCR to compare the recovery of polyadenylated viral RNA and vimentin mRNA versus 18S rRNA. While <5% of the highly abundant 18S rRNA was non-specifically pulled down, >10% and near 20% of viral RNA and poly(A) host transcripts were efficiently captured. These results confirm the selective enrichment of polyadenylated RNAs and support the reliability of our pulldown approach for isolating poly(A)-containing RNPs despite the presence of background rRNA (revised Figure 1F; Supplemental Figure 1B).　However, even if a mock-infected pulldown had been included, it would not exclude the possibility that proteins identified there could also bind viral transcripts upon infection. Thus, we emphasize that additional validation, such as RNA-IP, knockdown experiments, and functional assays, as performed in our study, is essential to confirm specific viral RNA-protein associations.

Minor comments:

1. Authors should read through the paper carefully as there are multiple spelling errors that should be fixed.

We thank the reviewer for pointing this out. We have thoroughly proofread the manuscript and corrected multiple typographical and spelling errors throughout the revised version.

2. The use of Reduction(%) in the axes in figures 7B and Supp Fig 2 and 3 should be replaced as it is not clear what is meant, and the legend does not clarify.

We agree that the previous labeling was unclear. In the revised Figure 4B, Figure 7B and 7D, Supplemental Figure 3B, and Supplemental Figure 4C, we have replaced 'Reduction (%)' with 'Relative protein levels.' As clarified in the revised figure legends (lines 1287-1292), protein levels in the mock or untreated condition were set to 100%, and values from experimental groups were normalized accordingly. This adjustment improves the clarity and interpretability of the quantitative data.

**Reviewer 3**

The manuscript by Lee et al., that identified SR proteins by metabolic labeling has been revised to address the reviewer comments. Overall, the authors have been more forthcoming about the limitations of their studies. This means that the short comings of the study are still present for the most part but now the reader will at least understand what they are. There are still significant issues with how they present their data and how well that is explained to the reader. Specifically, figure 1 shows that they have optimized some of their conditions for the pulldown such as the transcriptional inhibitor, but not others such as how clean the 4sU labeling and pull-down is. Given the preliminary data in figure 1, it seems clear that there will be a lot of non-specific proteins identified using their approach. Indeed, looking at Table S1 there are a lot of proteins that were identified. Curiously figure 2B shows the proteins that were at least two standard deviations above the mean suggesting these were the most vRNA associated proteins, but they didn’t really explore these, they went straight to the SRSF family of proteins, most of which were 1 standard deviation above the mean. Also, SRSF4 which was not identified by proteomics had a bigger effect on viral titers upon knockdown than most of the ones that were identified by mass spec. While one would not expect to have large effects on viral titers by knocking down a single RNA binding protein, SRSF3 had no affect while SRSF5 and 7 were only about 25% down, with SRSF4 and SRSF6 knockdown reducing titers by 40-50%. Also, it is noted that no cell viability data or control experiments showing that knockdown of these host proteins did not make the host cell less fit for viral amplification. The remainder of the manuscript focuses on finding a role for these SR or phospho-SR proteins. They look at co-localization with viral RNA, cell fractionation studies, polysome association, and IRES-mediated translation for which they observed a <2-fold change. This is on par with the KD experiments and similar to what others have seen when knocking down an RNA binding protein and performing either translation or plaque assays. Obviously, the authors have done a lot of new experiments to strengthen the manuscript since the initial review. The biggest issues are whether these host factors globally affect cell fitness for viral replication. This was not tested by using an unrelated virus such as a DNA virus. There is some worry that the claim of a pan antiviral target may just be due to inhibiting these proteins affects cell fitness for viral replication. The authors clearly worked to address the reviewers’ comments, however, there are still a number of issues with their experiments, experimental approaches, presentation of the data and conclusions that somehow leave the reader unsatisfied with their findings.

We sincerely thank the reviewer for the thoughtful and critical evaluation of our manuscript. We appreciate the acknowledgment of the additional experiments and revisions made to improve the clarity and transparency of our study. In this revised version, we have made a concerted effort to clearly present the limitations, improve figure labeling and resolution, and include additional controls and quantification to strengthen our conclusions. Below, we provide a detailed point-by-point response to address each of the reviewer’s remaining concerns.

**Major comments:**

1. In Figure 1F, it is unclear how much of the oligo(dT) and input are loaded on to the protein gel and how to think about this comparison. One would expect to see enhancement when 4sU was added for the oligo(dT) pull-downs but this doesn’t appear to be true for the 3CD and 3Dpol. The idea should be that leaving out the 4sU should not crosslink proteins so this would be the background. The viral proteins appear to be either the same or higher levels in the -4sU suggesting that the oligo(dT) pull-down is not clean.

We thank the reviewer for raising this important point. In the revised Figure 1 and its legend (lines 1241-1242), we have clarified the input and oligo(dT) loading volumes (2% of the input was loaded), which were not clearly labeled in the previous version. We agree that abundant proteins, such as 3CD and 3D^pol^, may non-specifically associate with oligo(dT) beads, especially at 8 hpi when viral protein levels are high. This potential background was noted in lines 202-205 of the revised manuscript, and to address this issue systematically, we performed quantitative mass spectrometry to compare protein enrichment between the 4sU-labeled and non-labeled conditions.

To rigorously evaluate enrichment, we applied log₂ transformation to the spectral count ratios. This approach helps normalize the distribution of proteomic data and minimizes the impact of highly abundant proteins. The rationale for using log₂ transformation is described in detail in our response to Point 4. This method allows for a more accurate and statistically robust identification of proteins specifically enriched in the 4sU condition.

2. Lines 185-6: “In contrast, proteins involved in genome replication were detected in the 8-h RNPs including the 3Dpol and hnRNPC (Figure 1F, lanes 6 and 8)” This statement doesn’t support what is observed Fig 1F. hnRNPC1/C2 is slightly higher with 4sU than without but this is very slight. What is more striking is how high it is in the 4 hpi with 4sU, which is not mentioned.

We appreciate the reviewer’s observation and agree that the abundance of hnRNPC1/C2 at 4 hpi is notable. We have revised the statement in the manuscript to better reflect the data shown in Figure 1F by stating: “While hnRNPC1/C2 is present at both time points, its prominent presence at 4 hpi may indicate additional roles beyond replication, such as in IRES-dependent translation regulation, as previously observed in cellular transcripts” (lines 198-200).

Although we have not investigated this further in the current study, our ongoing unpublished work suggests that the phosphorylation state of hnRNPC1/C2 may also be modulated during enterovirus infection. As this remains under investigation, we have limited our discussion to their previously reported role in host IRES-dependent translation.

3. Figure 1G: “RT-PCR analysis of the pull-down efficiency for RNA-protein complexes. Data show consistent results across at least three independent experiments.” Is this what should be expected? It seems to this reviewer that if oligo(dT) selected polyadenylated RNAs that there should have been increased ActB and vRNA in the oligo(dT) lanes compared to the input lanes which should be mostly rRNA. Where are the “at least three independent experiments?”. Lines 180-181: “RT-PCR analysis confirmed a comparable level of viral and host transcripts being pulled down (Figure 1G).” This review would expect that proof that the approach was working should result in more polyadenylated RNA in the pull-downs and less rRNA, which wasn’t shown.

We appreciate the reviewer’s insightful suggestion to include rRNA as a control to validate the specificity of the oligo(dT) pulldown. In the revised Figure 1F, we have now included quantitative PCR results for both viral RNA (vRNA) and 18S rRNA. Pulldown efficiency was evaluated by comparing to 5% of the input RNA as a reference. Our data show that while less than 5% of 18S rRNA was recovered in the pulldown, more than 10% or even 20% of vRNA was detected, indicating that the oligo(dT) pulldown effectively enriches polyadenylated transcripts, including viral RNA (lines 184-193). Although the absolute amount of rRNA remains high due to its natural abundance, the relative recovery rate supports the specificity of the pulldown.

Importantly, our conclusions are based on comparisons between the +4sU and -4sU conditions, which were processed in parallel under identical conditions. The rRNA detected in the pulldown may reflect low-level non-specific binding, possibly due to rRNA’s high abundance or indirect associations with poly(A) RNA via RNA-binding proteins. This further supports the need for quantitative mass spectrometry to identify proteins specifically enriched in the 4sU-labeled RNPs.

The results shown in Figures 1F and 1G were generated from the same control samples used in the mass spectrometry experiment. We have revised the Figure legend accordingly to clarify that these data serve as representative validation of both the assay system and the mass spectrometry workflow (lines 1238-1241).

4. Lines 194-6: “The average spectral count protein ratios between the 4sU and the non-4sU groups were calculated (a full list is provided in supplemental table S1). Based on the log2 mean value (-0.1973 and 0.0375 for the 4-h and 8-h RNPs, respectively)”. What is the purpose of the Log2 transformation? It seems to this reader to alter the data from being intuitive to something that is not. Why not just report the average spectral count ratios of the 4sU to the control. This would provide numbers above >1 as being enhanced and those at or below as not being enhanced. Then it’s just a matter of determining a reasonable cut off.

We appreciate the reviewer’s thoughtful comment. The purpose of the log₂ transformation was to stabilize variance and normalize spectral count ratios across a wide dynamic range, which is common in proteomic datasets. This transformation enables symmetric interpretation of fold changes (e.g., a 2-fold increase = +1; a 2-fold decrease = –1) and reduces the influence of outliers or highly abundant proteins.

While raw ratios may appear more intuitive, they can overrepresent non-specific background signals, as discussed in our response to Point 1. By using log₂ values, we were able to apply consistent and data-driven statistical thresholds (e.g., ±1 SD from the mean) to define enrichment or depletion more accurately. To clarify this approach for readers, we have added a brief explanation in the revised Methods section (lines 740-743).

5. Lines 311-313, the authors conclude from the cell fractionation and western analysis in figure 5 that: “Collectively, while the possibility of SR proteins regulating replication cannot be excluded, these findings suggest that RNPs enriched with phospho-SR proteins, including SRSF4, SRSF5, and SRSF6, may play significant roles beyond replication, potentially in processes such as translation.” The authors are correct they can’t rule out anything, but this reviewer has difficulty with the rest of the sentence as this data in figure 5 is not well controlled for the amount of protein loaded in each gel nor is there any significant changes that can be determined from these westerns. Also, the golgi lane for the pSR western seems to not be loaded equally as the background bands are noticeable fainter. Regardless this data would not even in its best state be able to speak as to whether these proteins play significant roles beyond replication… or in translation. Rather this conclusion seems to appropriately capture the data in figure 5 (line 310) “Notably, no obvious differences were observed in the infection group compared to the mock group.” Indeed, there is nothing striking presented in this data to shed further light on what, if any, role the SR or pSR proteins are doing during EVA71 infection.

We thank the reviewer for this thoughtful and detailed comment. We apologize for the insufficient description of our fractionation and loading strategy in the previous submission. To clarify, we collected organelle-enriched pellets by sequential centrifugation steps and used 10% TCA-precipitated lysate as the input control. All pellets were resuspended in the same volume of sample buffer as the input, and equal volumes were subjected to SDS-PAGE and western blotting (lines 1309-1312). This approach was intended to enable qualitative comparison of protein accumulation across different cellular compartments, assuming that total protein amounts across all fractions approximate the initial lysate.

As noted by the reviewer, the distribution of total SR proteins did not show obvious differences between mock and infected cells. However, we consistently observed that phospho-SR (pSR) proteins—especially SRSF4, SRSF5, and SRSF6—were enriched in fractions distinct from those containing viral replicase, suggesting possible association with non-replicative RNP complexes. We acknowledge that this conclusion was overstated in the previous version. We have revised the text accordingly (lines 342-347) to avoid overinterpretation and now describe their potential involvement in RNA processing events, rather than asserting a specific role in translation.

Regarding the faint pSR signal in the Golgi-enriched fraction at 8 hpi, we confirm that the pSR and SR blots were derived from the same membrane. The observed difference is therefore unlikely to result from uneven loading, and may instead reflect an overall decrease in pSR protein levels at this later time point (see Figure 4A). Raw data are available upon request. Nonetheless, we have revised the figure legend and text to clarify this point and improve the interpretability of the results (lines 1309-1312).

6. Figure 6C and lines 330-331 the authors conclude: “Western blot analysis revealed that the infection-specific hyper-phosphorylation state of SR proteins remained unaffected by either treatment (Figure 6C).” However, in the pSR EDTA lane, the banding pattern is distinctly different than in the other lanes.

We thank the reviewer for pointing this out and apologize for the oversimplified description in the previous submission. We have now revised the text to note the EDTA-induced hyper-phosphorylation, which is likely due to the inhibition of metal-dependent serine/threonine phosphatases (lines 364-367).

7. Lines 340-341” The authors state: “suggesting that the virus may redirect nuclear phospho-SR proteins to regulate translation during the early stages of infection.” The authors may want to be careful about over interpreting their findings as there are many interpretations for why the phosphor-SR proteins are polysome associated including that they are bound to the vRNA but not necessarily regulating its translation.

We greatly appreciate the reviewer’s thoughtful comment regarding the potential overinterpretation of our findings. In response, we have revised the conclusion in the text to emphasize the association between phospho-SR proteins and cytoplasmic viral RNA during infection, which may support IRES-dependent translation, rather than directly asserting a regulatory role (lines 373-377).

8. Figure 7D. It seems to this reviewer that a couple of things could be occurring. 1) that the pSR proteins somehow affect viral entry or the antiviral response but clearly the longer the inhibitors are present the larger the effect. It would have been nice to also see if washing out the drug would allow the authors to point to a specific window when the pSR proteins were required. In this way they could keep the amount time the cells were treated the same while probing specific times before, early or late in infection the drug inhibited titers. Also, showing that an unrelated virus, such as HSV or Ad, could replicate in the presence of this drug would be great. Cell viability assays can be less sensitive and problematic if they are not performed during the linear range.

We appreciate the reviewer’s thoughtful suggestions regarding Figure 7D (revised 7F). First, we acknowledge that performing a washout experiment could help better define the timing of pSR protein involvement during infection. However, this was not feasible in our current setup, as SRPKIN-1 acts through irreversible inhibition by forming covalent bonds with the target kinase. We have now clarified the irreversible nature of SRPKIN-1 in the revised manuscript (lines 385-389).

To address the reviewer’s concern about the timing of drug treatment, we have revised the time-of-addition assay schematic for better clarity and distinction of treatment windows (revised 7F). Accordingly, our data indicate that shorter treatment periods prior to infection (e.g., from –2 to –1 h or –1 to 0 h) resulted in a more pronounced reduction in viral titers compared to continuous treatment from 0 to 8 h post-infection. This suggests that the timing of drug addition may be more critical than the overall duration, potentially due to the irreversible action of the inhibitor.

We agree with the reviewer that testing the effect of the inhibitors on unrelated viruses would strengthen our conclusions. In response, we performed additional experiments using adenovirus (Ad) and herpes simplex virus (HSV), both DNA viruses (Rebuttal Figure 2). The virus strains and detailed assay procedures are described in the Materials and Methods section (lines 596, 606–607, 614–617, and 798–802). Our results (now included in Supplemental Figure 4D) show that these viruses also exhibited sensitivity to the inhibitors. This observation aligns with prior reports that pSR proteins are involved in the replication of DNA viruses via their roles in pre-mRNA splicing. We have added a brief discussion on this point in the revised manuscript (lines 383-385, 433-437).

Finally, regarding cell viability, we conducted a temporal analysis and now include these results in Supplemental Figure 4A (lines 389-391). While prolonged incubation with TG003 at 100 μM reduced viability to approximately 80%, we found that viability remained above 90% within a single viral replication cycle. This suggests that the observed antiviral effects are unlikely due to general cytotoxicity under the experimental conditions used.

**Rebuttal Figure 2. Inhibition of DNA virus replication by SR protein kinase inhibitors.** A plaque reduction assay was performed to assess the effects of combined treatment with TG003 (50 μM) and SRPKIN-1 (200 nM) on the replication of DNA viruses, including herpes simplex virus type 1 (HSV-1), herpes simplex virus type 2 (HSV-2), and adenovirus serotype 5 (Ad5). EV-A71 was included as a positive control. The plaque assay was conducted as described in the main text. Data are presented as the mean ± SD from three independent experiments. Statistical analysis was performed using the Student’s t-test. ****: p < 0.0001; ***: p < 0.001; **: p < 0.01..

9. Figure 7E. First the luciferase assays should not be normalized to a negative control, pRHF. Rather it should be normalized to a positive control such as pRHF-EV-A71 with viral infection set to 100%. This avoids issues with insignificant changes in the negative control having a profound effect on the significant numbers. Which strain of EVA71 was included in the reporter? Some have been shown to have cryptic promoter activity and therefore wouldn’t work in a DNA reporter. Have all the controls been performed for this plasmid? If so, include references as this data is not included. Figure S4 reveals the raw RLuc and Fluc values, however, ideally the raw Fluc values should look like the normalized data, but it does not rather the raw Fluc data appears to be unchanged from DMSO to the T+S drug combination. This would suggest that the change in the ratio is more due to changes in cap-dependent translation. It would have been more helpful to present the raw data for the n>3 biological replicates rather than a bar graph a single biological replicate with 3 technical replicates.

We thank the reviewer for these insightful comments and apologize for the lack of detail and clarity in our previous submission. We have verified the sequence integrity of our control and reporter plasmids based on the original publications. The IRES sequence used is derived from the EV-A71/2231/TW strain, as now stated in the revised Materials and Methods section (lines 886-888) and supported by references 30 and 56. Sequencing confirmation was performed to ensure construct fidelity; these data are available upon request.

We also agree that our previous normalization strategy using pRHF as the negative control could lead to misleading interpretations. In response, we have restructured our data presentation to first validate IRES activity independently, and then assess the effect of each drug treatment using DMSO as the baseline control. This revision helps minimize potential bias and better highlights changes due to drug treatment (lines 415-419).

Regarding Figure S4, we appreciate the reviewer’s observation. To enhance transparency, we have replaced the representative figure with the raw Firefly (Fluc) and Renilla (Rluc) luciferase values from three independent biological replicates, each performed in triplicate. These data are now provided in Supplemental Table S4. This addresses the concern that changes in the Fluc/RLuc ratio may reflect shifts in cap-dependent translation. The updated dataset and analysis now allow a clearer interpretation of how SR protein inhibitors impact IRES activity.

10. The authors should comment on why they did not see SRSF4 in their initial proteomics. Also, perhaps it was there if they looked a back at the data. If not, then why?

We thank the reviewer for highlighting the absence of SRSF4 in our initial proteomic data and acknowledge that this was not addressed clearly in our previous submission. Upon revisiting our datasets, we confirmed that peptides corresponding to SRSF4 were not detected in the input samples, and thus the protein was not quantified in the proteomic analysis. Given that triplicate samples were analyzed, this may reflect intrinsic limitations such as low abundance or the physicochemical properties of SRSF4-derived peptides, which may hinder their detection after trypsin digestion and MS analysis. Due to the potential variability in peptide detectability, affected by peptide abundance, digestion efficiency, and ionization properties, some proteins such as SRSF4 may not have been reliably detected, despite being present in the sample.

Although SRSF4 peptides were not detected in the input sample by mass spectrometry, SRSF4 was consistently observed as a prominent band in the vRNA immunoprecipitates using the pan-SR antibody. Additionally, we observed increased phosphorylation of SRSF4 upon EV-A71 infection (Figure 4A and 6C, Supplemental 3B and 4C), suggesting its potential involvement in infection-related processes. Based on these observations, we included SRSF4 in our siRNA screening, which revealed that its knockdown moderately reduced EV-A71 replication. These clarifications have been added to the revised manuscript (lines 277-281).

11. DNA and RNA transfections were not described in the methods.

We apologize for not providing sufficient details regarding the DNA and RNA transfection protocols in our previous submission. We have now included the amount of DNA/RNA used, the transfection reagent, and the cell density at the time of transfection in the Materials and Methods section (lines 888-891, 898-902).

**Minor comments:**

1. Figure 1E, I have no idea what the authors mean by pull-down efficiency.

We agree with the reviewer that using a semi-quantitative agarose gel is not an appropriate method to assess pull-down efficiency. To address this, we have replaced the data with quantitative RT-PCR analysis. As mentioned in response to Major point 3, we also included rRNA analysis to demonstrate the specificity of the pull-down (Figure 1F, lines 184-193).

2. Figure 1 legend needs to be specific about when the drug was added relative to the infection being performed. This is in Figures 1D,F but needs to be sooner in the legend assuming they were done the same. Also, it is noted that Figure S1 did not show a concentration range of ActD or the other inhibitors to determine optimal drug concentrations for each inhibitor. Also figure S1 used 4µM ActD while figure 1 used 5µM ActD.

We thank the reviewer for their careful observation. We have revised the figure legend to specify the timing of drug addition (Figure 1, lanes 1208-1209). All inhibitors were added during the virus adsorption step and maintained at the same concentration throughout the infection period.

As these transcription inhibitors are commonly used in virology studies, we selected concentrations based on the literature, particularly the review by Olivier Bensaude (Transcription, 2011, PMID: 21922053). Given our aim to achieve maximal inhibition of host transcription, we applied the highest suggested concentrations for each drug directly. We have now clarified the working concentrations and incubation times in the revised main text (lines 152-154).

Regarding the inconsistency in ActD concentrations between Figure 1 and Supplemental Figure S1, we apologize for the confusion. The 4 µM used in Figure 1 corresponds to 5 µg/ml, and this unit has been corrected in the revised supplemental figure legend and Materials and Methods section (lines 619, 1218-1219).

3. Figure 1B, the authors normalize the viral RNA to ActB transcript levels which seems odd given that Figure S1 shows ActB mRNA levels are decreasing with drug treatment as expected. One might expect the authors to normalize to an RNA that is not changing such as rRNA levels. Are the vRNA levels going up or is the ActB levels going down or both? Also, what does the Y-axis label mean Log10 100%?

We apologize for the confusion in our previous description. In this experiment, rather than normalizing to a housekeeping gene, we calculated viral RNA levels using ΔCt values relative to the initial RNA level immediately after viral adsorption (time 0), which was defined as 100%. This approach avoids confounding effects introduced by host transcription inhibitors, which also reduce *ActB* and other host mRNA levels, as shown in Figure S1. Additionally, fold change values from three independent experiments were used for statistical analysis to minimize the impact of potential handling variability.

To improve clarity, we have corrected the figure annotation: the Y-axis now reads “Fold change (log₁₀)” to reflect the log₁₀-transformed fold change relative to time 0. This change is also reflected in the revised figure legend (lines 1222-1225).

4. Figure 3E the authors need to specify the antibody or antibodies used.

We thank the reviewer for pointing this out. Each protein shown in Figure 3E was detected using a specific antibody against the corresponding SR protein. We have clarified this in the revised manuscript (lines 1269-1270), and detailed information for each antibody used is now included in the Materials and Methods section (lines 692-695).

5. Figure 3F what is mock? Is this no siRNA control? What is NC? These need to be defined in the legend. What time post infection were the plaque assays done? Also, was the virus harvested and then plaqued on fresh cells no knockdown or were the plaque assays performed on cells that were knocked down? This needs to be clear how the assay was done. If mock is no siRNA transfected it is a nice control as siRNA KD surely affects the interferon response of a cell.

We thank the reviewer for pointing out the need for clarification. In the revised figure legend (lines 1269-1271), we have now clearly defined "Mock" as cells without siRNA transfection and "NC" as cells transfected with a commercial negative control siRNA. Details of the experimental procedure are described in the Materials and Methods section (lines 771-782). Briefly, RD cells were transfected in suspension with each siRNA and plated onto 3.5 cm dishes. After 24 hours of incubation, the transfected cells were directly infected with the EV-A71 MP4 strain using a virus input calibrated to yield approximately 100 plaques per well after 48 hours, allowing for robust and countable plaque quantification. Following viral adsorption, an agarose-containing overlay was applied, and plaque numbers were counted after 48 hours. This setup enabled us to evaluate the direct impact of each siRNA on EV-A71 replication.

To rule out cytotoxic effects caused by siRNA transfection, we also performed a cell viability assay. While knockdown of SRSF3 and SRSF5 slightly reduced RD cell viability, no significant cytotoxicity was observed in cells transfected with other siRNAs after 72 hours of incubation (Supplemental Figure 2, lines 270-272). These results confirm that the observed decrease in plaque formation upon SRSF4, SRSF5, SRSF6, and SRSF7 knockdown was not due to cytotoxicity, but rather reflects a specific role of these SR proteins in viral replication.

6. Figure 4D It is not at high enough magnification or the figure is not at high enough resolution to see nuclear speckles. Also the cytoplasmic localization of the SR proteins is not clear at this magnification. However, the pSR cytoplasmic localization following infection is clear.

We thank the reviewer for pointing out the limited visibility of nuclear speckles and cytoplasmic localization of SR proteins in Figure 4D. All images in Figure 4 were captured using a confocal microscope with a 60× oil immersion objective, as stated in the Materials and Methods section (lines 814-816). To enhance the visualization of both nuclear speckles and the faint cytoplasmic signal of SR proteins, we recalibrated the fluorescence signal intensity. Additionally, we selected representative regions enriched in cells showing cytoplasmic relocalization of SR proteins, rather than presenting a raw wide-field view, to better illustrate the phenomenon. In the revised Figure 4C, we have included a zoomed-in view to improve clarity, and we will ensure that the final image resolution supports the visualization of punctate nuclear speckles during submission.

7. Figure 4E: is this still at an MOI of 40 like was done in figure 4D? If so wouldn’t one expect the SR proteins to relocalize to the replication complexes more than half of the time?

We thank the reviewer for the insightful comment. Although an MOI of 40 is typically considered sufficient to achieve high infection rates, in our experience, even when using antibodies against viral proteins to identify infected cells, the proportion of positive cells rarely exceeds 50% at early time points. This observation aligns with findings from Guo et al. (*Cell Rep*, 2017, PMID: 29117571), who used single-cell analysis to demonstrate cell-to-cell variability in susceptibility and replication dynamics during poliovirus infection.

Due to the use of antibodies of the same isotype in this study, we were unable to perform double staining to simultaneously detect viral proteins and SR/pSR proteins. Nevertheless, the quantified data in revised Figure 4C and 4D are consistent with previous staining results using viral protein antibodies. In addition, we observed that the proportion of cells with cytoplasmic SR/pSR increased at later time points post-infection (data available upon request).

Furthermore, RNA-FISH analysis in the revised Figure 4E and 4F provides additional support for this conclusion, showing that only a subset of cells were positive for viral RNA, indicating active replication. We have clarified this point in the revised manuscript (lines 311-312). We have also combined the images and their corresponding quantification results into the revised Figure 4C and 4D to present the data for SR and pSR proteins, respectively.

8. Figure 4G, was this confocal microscopy? This needs to be clarified.

We thank the reviewer for the helpful comment. We confirm that all images, including those shown in Figure 4G, were acquired using confocal microscopy, as described in the Materials and Methods section (lines 814-816, 824-825). This has also been clarified in the revised figure legend (lines 1304-1305).

9. Figure 7H should be re-labeled 7G.

We apologize for the labeling oversight. The figure has been corrected and we have carefully reviewed the revised manuscript to ensure consistency across all figure labels.
